# *PIGG* defines the Emm blood group system

**DOI:** 10.1038/s41598-021-98090-w

**Published:** 2021-09-17

**Authors:** William J. Lane, Judith Aeschlimann, Sunitha Vege, Christine Lomas-Francis, Anna Burgos, Helen H. Mah, Justin B. L. Halls, Peter Baeck, Peter C. Ligthart, Barbera Veldhuisen, Ripal J. Shah, Sanmukh R. Joshi, Connie M. Westhoff

**Affiliations:** 1 Department of Pathology, Hale Building for Transformative Medicine, Brigham and Women’s Hospital, Rm 8002L, 60 Fenwood Rd, Boston, MA 02115 USA; 2 Harvard Medical School, Boston, MA USA; 3 Immunohematology and Genomics Laboratory, New York Blood Center, New York, NY USA; 4 Childrens Clinic, County Hospital, Kalmar, Sweden; 5 Department of Immunohematology Diagnostic Services, Sanquin, Amsterdam, The Netherlands; 6 Prathama Blood Center, Ahmedabad, India; 7 Department of Research, Lok Samarpan Raktdan Kendra, Surat, Gujarat India

**Keywords:** Genetics research, Molecular medicine, Personalized medicine, Erythropoiesis

## Abstract

Emm is a high incidence red cell antigen with eight previously reported Emm− probands. Anti-Emm appears to be naturally occurring yet responsible for a clinically significant acute hemolytic transfusion reaction. Previous work suggests that Emm is located on a GPI-anchored protein, but the antigenic epitope and genetic basis have been elusive. We investigated samples from a South Asian Indian family with two Emm− brothers by whole genome sequencing (WGS). Additionally, samples from four unrelated Emm− individuals were investigated for variants in the candidate gene. Filtering for homozygous variants found in the Emm− brothers and by gnomAD frequency of < 0.001 resulted in 1818 variants with one of high impact; a 2-bp deletion causing a frameshift and premature stop codon in *PIGG* [NM_001127178.3:c.2624_2625delTA, p.(Leu875*), rs771819481]. *PIGG* encodes for a transferase, GPI-ethanolaminephosphate transferase II, which adds ethanolamine phosphate (EtNP) to the second mannose in a GPI-anchor. The four additional unrelated Emm− individuals had various *PIGG* mutations; deletion of Exons 2–3, deletion of Exons 7–9, insertion/deletion (indel) in Exon 3, and new stop codon in Exon 5. The Emm− phenotype is associated with a rare deficiency of *PIGG*, potentially defining a new Emm blood group system composed of EtNP bound to mannose, part of the GPI-anchor. The results are consistent with the known PI-linked association of the Emm antigen, and may explain the production of the antibody in the absence of RBC transfusion. Any association with neurologic phenotypes requires further research.

## Introduction

The Emm red blood cell (RBC) antigen is assigned number 901008 in the International Society of Blood Transfusion (ISBT) 901 series of high incidence antigens^1,2^. Anti-Emm was first observed in 1973, and reported in 1987 in four unrelated individuals who had a broadly reactive RBC alloantibody that was mutually compatible between the probands^3^. To date, a total of eight Emm− probands of various nationalities are known. The majority were males presenting with IgM and/or IgG antibodies in the absence of exposure to allogeneic RBCs^3–6^. A clinically significant acute hemolytic transfusion reaction was reported in the one individual who was transfused^5^.

Anti-Emm does not react with RBCs from individuals with paroxysmal nocturnal hemoglobinuria (PNH) type III, which lack glycosylphosphatidylinositol (GPI) anchored proteins, principally due to somatic mutation in phosphatidylinositol glycan A (*PIGA*)^7^. This observation suggested that Emm is located on a GPI-linked protein^4,8^. We previously found by Western blot and hemagglutination that RBCs from Emm− individuals do not lack GPI-anchored acetylcholinesterase (*ACHE*, Yt blood group), complement decay-accelerating factor (*CD55*, Cromer blood group), ecto-ADP-ribosyltransferase 4 (*ART4*, Dombrock blood group), lymphocyte function-associated antigen 3 (*CD58*), and CD59 glycoprotein (*CD59*, CD59 blood group)^4^.

More than 150 proteins are GPI-anchored via post-translational modification in the endoplasmic reticulum (ER). GPI-anchor biosynthesis requires 27 known genes^7^, with the synthesis of the GPI-anchor alone involving 11 steps and over 15 genes^7,9^. GPI-deficiency has been reported due to recessive defects in 21 of the 27 genes involved in the GPI-anchor pathway^7^. These include the numerous *PIG* genes which are involved in the biosynthesis of GPI (phosphatidylinositol glycans) (*PIGA-PIGZ)*, and the *PGAP* genes involved in modification of the GPI anchor. There are reports of neurological phenotypes including seizures, developmental delay/intellectual disability, cerebral atrophy and hypotonia associated with mutations in genes involved in the GPI anchor pathway^7,10^.

Here we report the serologic and molecular investigation and whole genome sequencing (WGS) of samples from a South Asian family in which two brothers were found to be Emm− with anti-Emm present in the plasma. The apparent genetic cause for the Emm− phenotype in this family was found to be a 2-bp deletion in the *PIGG* gene which causes a frameshift and predicted premature stop codon [c.2624_2625delTA, p.(Leu875*)]. *PIGG* encodes an enzyme, ethanolamine phosphate transferase 2, that modifies the second mannose of the GPI-anchor by adding ethanolamine phosphate (EtNP). *PIGG* deletion mutations were also present in samples from two additional unrelated probands of Japanese and European extraction previously investigated in our laboratory, and in two generations of a North African family with two sisters who also had Emm− RBC phenotypes with anti-Emm identified during pregnancy. Mutations included homozygous deletion of *PIGG* Exons 2 and 3, deletion of Exons 7 thru 9, and insertion deletion in Exon 3. Lastly, two siblings reported previously and known to have loss of function *PIGG* mutations^11^ were found to have Emm− RBC phenotypes.

## Methods

### Human participant statement

All methods were carried out in accordance with relevant guidelines and regulations. Serologic typing and antibody identification had been previously performed as part of routine clinical testing. All other experiential protocols including targeted sequencing and WGS were performed on archived samples with approval from the Mass General Brigham HealthCare Human Research Committee (IRB), which is the umbrella organization IRB that oversees the Brigham and Women’s Hospital. The IRB approved access to previous clinical results and for new experiments on archived samples under an excess clinical sample protocol, which was deemed to be minimal risk given no direct patient involvement and thus was exempt from obtaining informed consent.

### Samples and serologic reactivity of anti-Emm

Blood samples were collected in EDTA. RBC antigen typing and antibody identification was performed by standard tube methods^12^. Genomic DNA was isolated by standard methods (QIAamp, QIAGEN, Inc. Valencia, CA) from fresh samples and frozen RBC samples.

Briefly, interaction between antibody and red cells is observed as agglutination and often requires use of a secondary anti-human globulin reagent. Serum or plasma containing the antibody and red cells of known phenotypes are incubated at 37 °C, centrifuged, and examined for agglutination. For the indirect antiglobulin test (IAT), following incubation the cells are washed with phosphate buffered saline to remove unbound immunoglobulins, and antihuman globulin is added, centrifuged, and examined for agglutination. For in vitro enhancement of the interaction of red cell antigens and antibodies, low ionic strength solution (LISS) or Polyethylene glycol (PEG) are added prior to incubation. Treating the test cells with proteolytic enzymes such as papain, ficin, or trypsin can be used to enhance antibody-antigen reactions or, in addition to dithiothreitol (DTT), can be used to identify the specific antibody target based on known sensitivity or cleavage pattern of the red cell protein.

Proband 1, a 65-year-old group AB, D+ Indian male with heart disease, presented with an antibody that reacted with all panel cells tested but did not react with autologous RBCs. The reactivity in saline at 4 °C and by the IAT with LISS enhancement was 2+, with 3+ reactivity by IgG gel test. The antigen being detected was resistant to treatment with papain, trypsin α-chymotrypsin and dithiothreitol (DTT); papain treatment enhanced the reactivity. Testing of the RBCs with antibodies to high prevalence antigens from our collections showed that his RBCs were Emm–. His plasma was nonreactive with three examples of Emm– RBCs from our collections: two Caucasian U.S. patients^4^ and one from the Japanese Red Cross^5^. Testing of the family revealed a compatible younger brother whose RBCs were Emm– and although he too had never been transfused, his plasma contained anti-Emm (1+ by IgG gel). RBCs of the proband’s daughter, his son, and wife were Emm+ and incompatible when tested with the proband’s plasma.

DNA was extracted from stored RBCs of Proband 2, who was previously reported in abstract form in 2013^5^. This 58-year-old Japanese man with total blindness and renal carcinoma, who had never been previously transfused, was in urgent need of transfusion due to massive bleeding. An antibody reacting 2+ with all cells tested was detected in the plasma, but his clinical condition required transfusion of crossmatch-incompatible blood. He experienced an acute hemolytic transfusion reaction (HTR); his RBCs reacted in the DAT: 1+ on day 1, 2+ on day 3, and negative on day 7, suggesting complete removal of the transfused RBCs from circulation. The antibody was identified as anti-Emm and was shown to have both IgG1 and IgG3 components and to fix complement. The titer of the antibody increased from 16 (saline-IAT) pre-transfusion to 128 by day 10.

DNA was extracted from stored RBCs of Proband 3, who was previously reported by our laboratory in an abstract in 1998^4^. This 70-year-old untransfused male of European ancestry was admitted for transurethral resection of the prostate (TURP) and, like other Emm– probands presented with an antibody that was reactive with all cells tested but nonreactive with autologous RBCs. The antibody reactivity was strong: 3+ to 4+ by albumin and PEG IAT and 4+ when tested against enzyme treated and DTT treated RBCs.

Proband 4 and her family are of North African origin^6^. In 2012 this 26-year-old female presented at week 25 of her first pregnancy with an antibody against a high frequency antigen resistant to trypsin, ficin, DTT, chymotrypsin and AET. The antibody reacted 2+ in the PEG IAT, and weakly positive in the LISS gel technique, with a negative auto control. The antibody was identified as anti-Emm and her RBCs were Emm−. She had never received a blood transfusion. Her plasma was incompatible with RBCs from her husband, parents, and six of her seven siblings. One sister was found to be compatible and was also Emm−.

Proband 5 and her brother are of Palestine origin, previously reported to have a loss of function *PIGG* variant and nonprogressive severe generalized ataxia and tonic clonic seizures with moderate delayed development^11^, were also investigated. Neither child had ever been transfused. Their RBCs typed as Emm−. Anti-Emm was not detected in the plasma.

### Illumina short read WGS

PCR free Illumina paired-end short read WGS was performed using standard methods. Briefly, genomic DNA (350 ng in 50µL) was fragmented acoustically using a Covaris focused-ultrasonicator (385 bp fragments). Additional size selection was performed using solid-phase reversible immobilization. Library preparation was performed using a commercially available kit (KAPA Biosystems, Wilmington, MA. Hyper Prep, product KK8505), and with palindromic forked adapters with unique 8-base index sequences embedded within the adapter (Integrated DNA Technologies, Coralville, IA). Libraries were quantified using quantitative PCR (KAPA Biosystems) using Agilent’s automated Bravo liquid handling platform. Libraries were normalized to 1.7 nM and pooled into 24-plexes. Sample pools were combined with HiSeqX Cluster Amp Reagents EPX1, EPX2 and EPX3 into single wells on a strip tube using the Hamilton Starlet Liquid Handling system. Cluster amplification was performed according to the manufacturer’s protocol (Illumina, San Diego, CA) with the Illumina cBot. Flow cells were sequenced on Illumina HiSeqX utilizing sequencing-by-synthesis kits to produce 151 bp paired-end reads. Output from Illumina software was processed by the Picard data-processing pipeline^13^ to yield CRAM files^14^ containing demultiplexed, aggregated aligned reads referenced to GRCh38/hg38. The Integrative Genomics Viewer (IGV)^15^ was used as needed to verify sequence identity and depth of coverage in the CRAM file (i.e. the number of times a specific site was sequenced). The CRAM files were genotyped using Genome Analysis Tool Kit (GATK) HaplotypeCaller v3.5-0-g36282e4^16^ to create variant calling format (VCF) files containing germline short variant calls (SNVs [single nucleotide variations] and indels [insertions and deletions]) found in the samples.

### Identification of potential variants

The VCF files were annotated with gnomAD v3^17^ allele frequencies using vcfanno v0.3.2^18^ (variants not present in gnomAD were inferred to have an allele frequency of 0). A custom program was written in Go v1.15.2^19^ to filter the annotated VCF to only keep homozygous variants found in the Emm− proband and his brother, but not homozygous in the proband’s two children. The VCF file was then filtered to only include variants with a gnomAD allele frequency < 0.001. The potential impact of the remaining variants were scored by Ensembl Variant Effect Predictor (VEP) v101^20^ (–sift b –regulatory –hgvs options). The following *PIGG* reference sequences were used throughout the manuscript: NG_051621.1 (genomic) and NM_001127178.3 (transcript).

### Sanger sequencing

*PIGG* Exons 1–13 were amplified (HotStarTaq Master Mix Qiagen, Hilden, Germany) using primers (Table S1) with the following PCR profile; 15 min at 95 °C, 35 cycles of 30 s at 94 °C, 30 s at 58 °C and 30 s at 72 °C, and a final elongation of 10 min at 72 °C. Long range amplification was performed (PrimeSTAR GXL, Takara Bio, Madison, WI) with 30 cycles of 10 s at 98 °C and 3 min at 68 °C for *PIGG* Exons 1 to 4 and 35 cycles of 10 s at 98 °C and 4 min at 68 °C for *PIGG* Exons 6–10. Products were visualized by agarose gel electrophoresis and ethidium bromide staining and purified with ExoSAP-IT (Applied Biosystems, Foster City, CA). Sanger sequencing was performed and analyzed with ClustalX.

### Targeted next generation sequencing (NGS)

Long range amplified products (8 ng at 1.6 ng/uL) were subjected to NGS library preparation using the Illumina Nextera XT kit (Illumina, San Diego, CA) according to the manufacturer's instructions. Sequencing was performed on the Illumina MiSeq platform (Illumina, San Diego, CA) to generate 150 bp paired-end sequence reads using a nano flow cell (Illumina, San Diego, CA). Sequencing reads were aligned to GRCh38/hg38 human reference genome using BWA-MEM v0.7.12-r1039^21^. The resulting SAM file was then transformed through the use of a series of steps: samblaster v0.1.24^22^ to addMateTags and removeDups, samtools v1.7^23^ to convert to BAM and sort file by genomic coordinates, Picard v2.5.0^13^ to AddOrReplaceReadGroups, and then samtools v1.7^23^ to create BAM index file. The Integrative Genomics Viewer (IGV)^15^ was used to view the sequence depth of coverage and paired sequence reads.

## Results

### Variant identification

**Figure 1:**
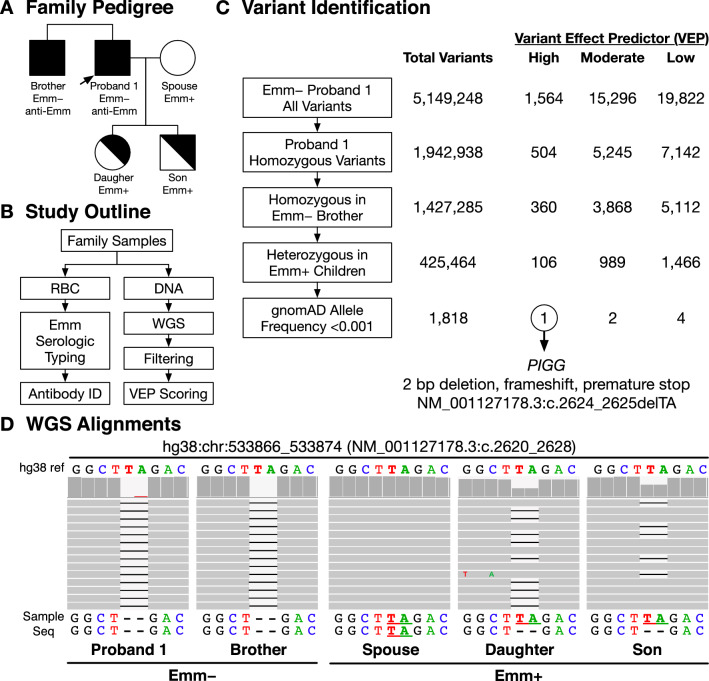
(**A**) Family Pedigree. Emm RBC phenotypes shown include the Proband and his brother (both with anti-Emm in the plasma). Also shown are the Proband's spouse and two children who are Emm+ and were shown to lack the antibody. (**B**) Study Outline. Emm serologic RBC typing was performed with in-house reagents and short read whole genome sequencing (WGS). (**C**) Variant Identification. Illustration of the strategy used to enrich for loss of function mutations associated with familial inheritance and variant effector prediction (VEP) scoring to identify the genetic cause of the Emm− phenotype. *PIGG* was the only candidate gene that passed our filtering strategy. (**D**) WGS alignments. IGV genomics viewer shows the wild type sequence (upper) and *PIGG* Exon 12 biallelic sequence for each family member (below). Individual sequence reads are shown in gray for positions corresponding to the hg38 reference sequence with bar plots above (dark grey) reflecting the relative number of reads at that position. Emm− family members were homozygous for a 2-bp deletion in Exon 12, predicted to cause a frameshift and premature stop, designated in as c.2624_2625delTA, p.(Leu875*), rs771819481, and chromosomal location hg38:chr4:533870_533871delTA. The spouse was wild type, and the daughter and son are heterozygous.

Figure 1A shows the pedigree of the South Asian family of Proband 1 and the RBC phenotypes including his brother, spouse, and children. Figure 1B shows the approach to identify the genetic variants (SNVs and indels) that might be associated with the Emm− phenotype. Variants present in WGS were enriched through a series of filtration criteria that included the prediction of the impact of the genomic change as none, low, moderate, or high using VEP (Fig. 1C). Initial analysis identified 5,149,248 variants in the Emm− proband, with 1,942,938 homozygous, including 504 of predicted high impact. Variants were then filtered for homozygous variants present in both the Emm− proband and his brother which left 1,427,285 variants with 360 of high impact. Filtering for those heterozygous in his Emm+ children reduced the number of variants to 425,464 with 106 of high impact. The remaining variants were then filtered for variants either not present in the gnomAD variant database or with a frequency < 0.001 which resulted in 1,818 with 1 predicted to have a high impact. The high impact variant was a 2-bp deletion in Exon 12 of the *PIGG* gene, which results in a frameshift and predicted premature stop codon designated c.2624_2625delTA, p.(Leu875*) in the cDNA (rs771819481) with chromosomal location hg38:chr4:533870_533871delTA. Figure 1D shows the reference sequence and biallelic WGS of the affected region of Exon 12 for each family member. The Emm− brothers were homozygous, the spouse was wild type, and daughter and son were heterozygous.

**Figure 2:**
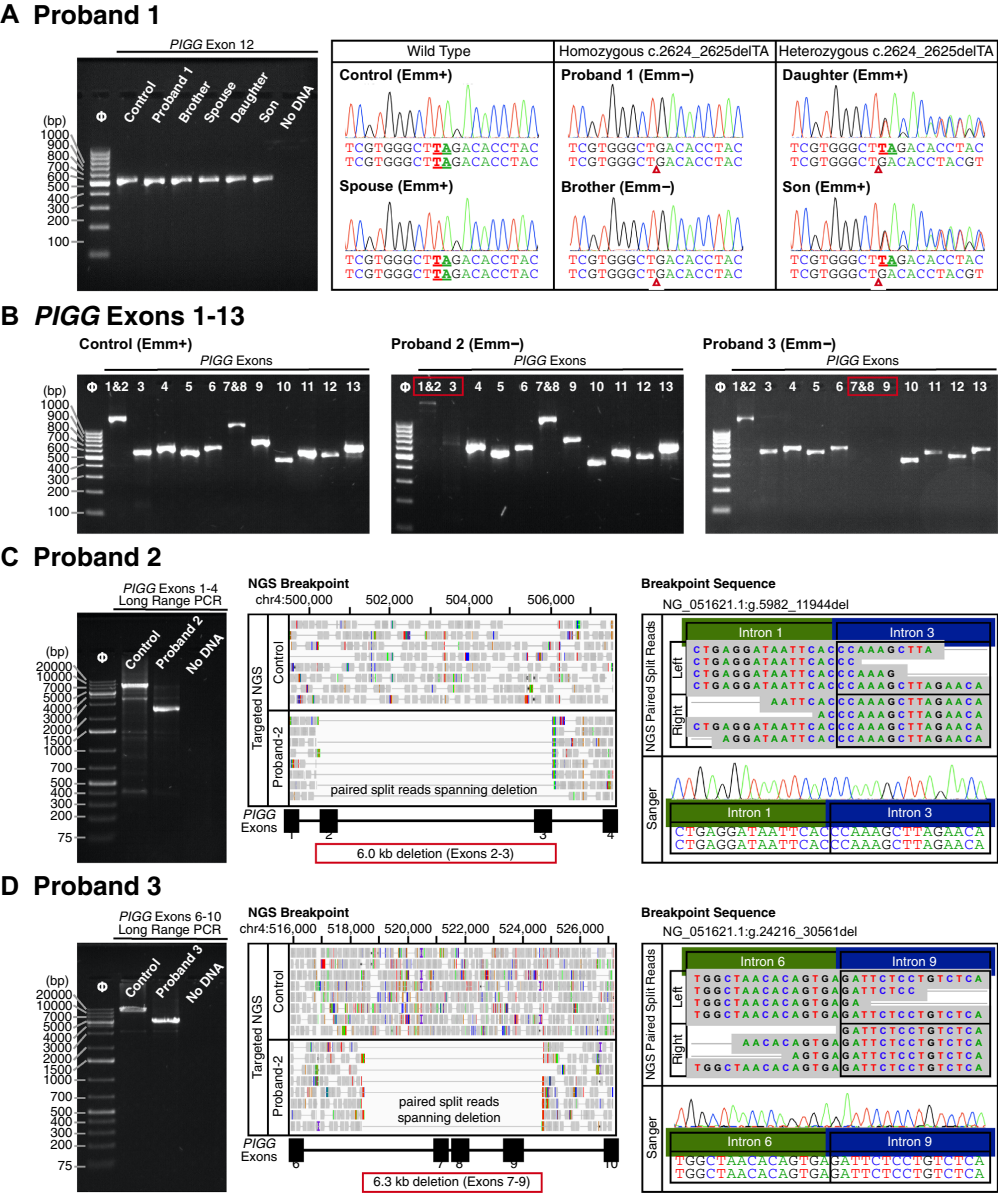
(**A**) *PIGG* Exon 12. Products ~ 506 bp were amplified from a Emm+ control sample and the family of Proband 1 (left) and Sanger sequencing (right) confirmed the homozygous 2-bp deletion in the Emm− brothers, the wild type sequence of the spouse, and heterozygosity for the daughter and son. (**B**) Amplification of *PIGG* Exons 1 through 13. Amplified products covering the 13 Exons are shown. Exons 1 & 2 and 7 & 8 were designed to be amplified as a single product. Exons 2–3 did not amplify in the sample from the Japanese Proband 2 and Exons 7–9 did not amplify in that from the European Proband 3. (**C**) Proband 2 deletion breakpoints. Amplification of Exons 1–4 in the sample from the Japanese Proband 2 indicate an approximate 6.0 kb deletion in *PIGG* compared to the control (left). Targeted NGS indicated a breakpoint spanning the region of Exons 2 and 3 compared to control (middle). Analysis of paired split reads aligned to both the left and right side of the deletion [g.5982_11944del, p.(Ala53Glyfs*2)] showed reads spanning the breakpoint in Intron 1 and Intron 3 (representative reads shown), and Sanger sequencing confirmed the breakpoint sequence (right). (**D**) Proband 3 deletion breakpoints*.* Amplification spanning Exons 6–10 in a sample from the European Proband 3 indicates an approximate 6.3 kb deletion in *PIGG* compared to the control (left). Targeted NGS indicated a breakpoint spanning the region of Exons 7 through 9 (middle). Analysis of the paired split reads aligned to both the left and right side of the deletion [g.24216_30561del, p.(Asp372Alafs*18)]] showed reads spanning the breakpoint in Intron 6 and Intron 9 (representative reads shown), and Sanger sequencing confirmed the breakpoint sequence (right).

Primers were designed (Table S1) to amplify and Sanger sequence all *PIGG* Exons to confirm the WGS results. Figure 2A shows the amplification and Sanger sequence results for Exon 12 which confirmed the absence of a deletion in a Emm+ control and in the spouse, homozygosity for the 2-bp deletion in the Emm− proband and his brother, and heterozygosity in the two Emm+ children.

### *PIGG* mutations in additional Emm− probands and breakpoint analysis

Samples from two unrelated Emm− probands from our collections and from two generations of a North African family, consisting of 10 individuals, underwent genomic investigation by Sanger sequencing and targeted NGS of *PIGG*. Figure 2B shows the amplification products for the 13 *PIGG* Exons in a Emm+ control, and from Proband 2 and Proband 3. *PIGG* Exon 3 failed to amplify in Proband 2 and the weak product from Exon 1 and 2 suggested Exon 1 and/or 2 involvement. For Proband 3, Exons 7, 8, and 9 failed to amplify.

Long range amplification targeting Exons 1 and 4 in Proband 2’s sample (Fig. 2C) compared to a control confirmed the presence of a deletion in *PIGG*. Sequencing of the Exon 1–4 product using targeted NGS found a deletion of 6.0 kb spanning Exons 2 and 3 and identified breakpoint regions in Intron 1 and 3 [g.5982_11944del, p.(Ala53Glyfs*2)]. Sanger sequencing confirmed the specific breakpoint sequence (Fig. 2C).

Long range amplification targeting Exons 6 and 10 in the sample from Proband 3 (Fig. 2D) and compared to a control confirmed the presence of a deletion in *PIGG*. Targeted NGS of the product identified a 6.3 kb deletion spanning Exons 7, 8, and 9 and located the breakpoint regions in Intron 6 and 9 [g.24216_30561del, p.(Asp372Alafs*18)]. Sanger sequencing confirmed the specific breakpoint sequence (Fig. 2D).

**Figure 3:**
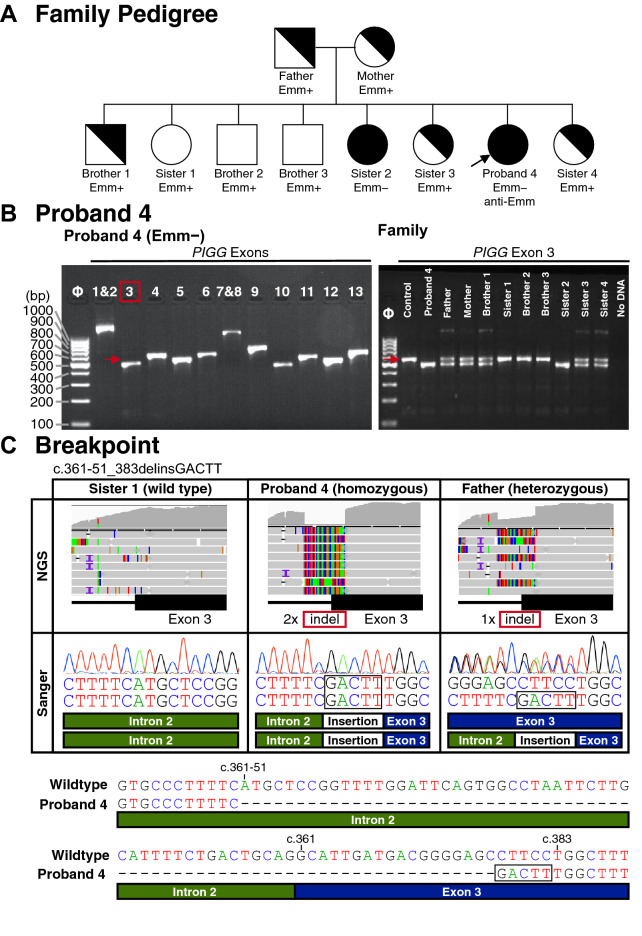
(**A**) Family Pedigree Proband 4. Emm RBC phenotypes shown include the North African Proband, who was pregnant with anti-Emm in the plasma, and her parents and 7 siblings. (**B**) Amplification of *PIGG* Exons 1 through 13. Amplified products covering the 13 Exons are shown. Arrow indicates the location of the Exon 3 product in the control sample. Exon 3 demonstrates ~ 100-bp deletion in the sample from Proband 4 compared to control. (**C**) Proband 4 deletion breakpoints. Amplification of Exon 3 in the sample from North African Proband 4 indicated an approximate insertion deletion (indel) in *PIGG* compared to the control (left). Targeted NGS indicated a breakpoint spanning the region of Intron 2 and Exon 3 compared (top). Analysis showed split reads containing the breakpoint sequencing spanning the deletion in Intron 2 and Exon 3 with 5 extra bases inserted near exon 3 (colored rectangles in alignment), c.361-51_383delinsGACTT, p.(Ala121_Pro128delinsAspPhe). Breakpoint was confirmed by Sanger sequencing (middle, note Sanger traces are reverse complemented since sequencing done using reverse primer). Deletion breakpoint sequence compared to wild type (bottom).

Figure 3A shows the pedigree of the North African family of Proband 4 and the RBC phenotypes of her parents, three brothers, and four sisters. Amplification of *PIGG* Exons 1–13 from genomic DNA from the proband found all Exons were present (Fig. 3B left). However, amplification of Exon 3 resulted in a smaller product compared to control in the proband and her Emm− sister (Fig. 3B right) and suggested the presence of a deletion in Exon 3 in *PIGG* as the cause of the Emm− phenotype in this family. The results identified the parents as potential heterozygote carriers along with three of the seven siblings, and revealed the three wild-type individuals in the family. Products obtained by PCR amplification of Exon 3 were sequenced by both targeted NGS and Sanger and showed the Emm− phenotype was associated with homozygosity for a deletion/insertion (indel) event with deletion of 74 bp consisting of 51 bp of Intron 2 and the first 23 nucleotides of Exon 3, along with insertion of 5 bp, GACTT (Fig. 3C), designated c.361-51_383delinsGACTT, p.(Ala121_Pro128delinsAspPhe). Sanger sequencing confirmed the specific breakpoint sequence.

RBCs from Proband 5 and her brother, previously reported to have a *PIGG* loss of function variant [c.1640G > A, p.(Trp547*), rs547951371] identified by WES^11^, were found to be Emm− and Sanger sequencing confirmed the variation.

### Rare *PIGG* mutations and Emm− phenotypes

**Figure 4:**
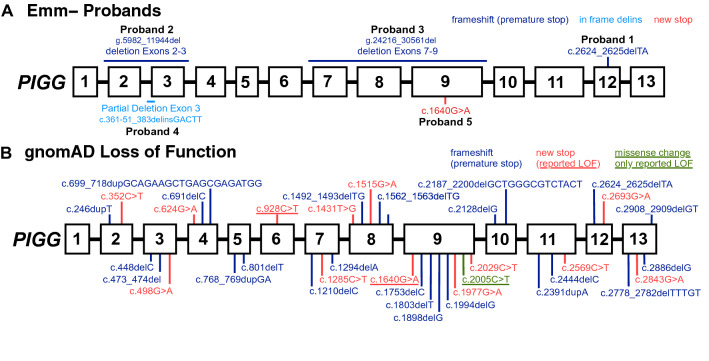
*PIGG* loss of function variants. (**A**) The five loss of function variants reported or investigated here are shown. Three causes a frameshift and premature stop codon, one causes a deletion and insertion, and one introduces a stop codon. (**B**) *PIGG* loss of function variants found in gnomAD cause frameshift and premature stop codons (blue) or directly introduce a stop (red). Previously reported loss of function variants associated with neurologic phenotypes are shown underlined, including variants that alter splicing (purple) and missense variants (green).

Figure 4A illustrates the five different *PIGG* mutations associated with the Emm− phenotypes reported here. The *PIGG* 2-bp deletion mutation, c.2624_2625delTA, was found in gnomAD v3 with an allele frequency of 0.000014 (2/143,344 alleles; query 4-533,869-TTA-T, Table S2) also in two heterozygotes South Asians (2/3,052 alleles; 0.0006553 frequency), the same ethnicity as Proband 1 who was homozygous for this mutation. The *PIGG* stop codon found in Proband 5, c.1640G > A was found in one heterozygous Non-Finish European in gnomAD (1/143,298 alleles; 0.000007 frequency). gnomAD SVs v2.1 contains no deletions paralleling those found in the probands here, nor large deletions encompassing other regions of *PIGG*.

Figure 4B (and Table S2) summarizes 37 rare variants with the potential for *PIGG* loss of function found in gnomAD v3: 13 are missense variants introducing a stop codon, and 24 are frameshift variants predicting premature stop codons. Most of the possible loss of function *PIGG* variants were found in only a single individual, 29 of 37 variants. A notable exception is c.1515G > A [p.(Trp505Ter), rs150259543] found in 102 /143,304 alleles, with a frequency of 0.000712. A total of 97 of the 102 alleles are found in Non-Finnish Europeans, and the c.1515G > A change is the only variant with a homozygous individual currently in the database. Structural variants (SV) found include two rare partial *PIGG* duplications (0.0002 and 0.00005 frequency), which might lead to loss of function. Overall, the allele frequency of *PIGG* loss of function is 0.00107 (153/143,364 alleles, or 1/937 individuals), which predicts a homozygous Emm− prevalence of 0.00000114, or 1/877,969 individuals.

### GPI anchor biosynthesis and the Emm antigen

**Figure 5:**
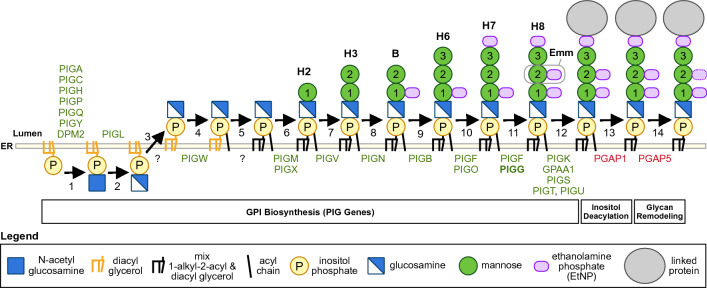
GPI anchor synthesis pathway. The schematic shows the relevant part of the GPI anchor synthesis pathway. The proteins involved in each step are indicated below the line. PIGG transferase (bold) is reported to be expressed during erythropoiesis, but loss of function in Emm− individuals would mean EtNP is not added to mannose 2. EtNP on mannose 2 is the likely antigenic target responsible for anti-Emm.

Figure 5 illustrates steps in the complex GPI anchor biosynthesis pathway in mammalian cells. GPI-anchoring is a post-translational modification, with the core GPI assembled on the endoplasmic reticulum (ER) membrane. Building of the GPI-anchor begins with inositol phosphate to which glycans are added, including glucosamine and three mannose residues (Man1, 2, 3), along with the addition of ethanolamine phosphate (EtNP) side chains^7,9^. *PIGG* along with *PIGF* encode the GPI-ethanolamine transferase II (GPI-ETII) enzymatic complex which adds EtNP to Man2, converting the GPI precursor H7 to H8 (Fig. 5)^24^. *PIGG* loss of function in Emm− individuals would cause loss of addition of EtNP to the second mannose residue (Man2) in the GPI link (shown on H8). The protein is then linked to the GPI-anchor, with remodeling by PGAP1, which removes the acyl chain linked to inositol, and PGA5 which removes some of the EtNP on Man2. This remodeling is thought to regulate transport to the Golgi in some cell types^11,25^. However, it has been reported that RBCs keep the acyl chain removed in other cells by the deacetylase PGAP1^7^. To gain additional insight into the structure of the potential target of anti-Emm, we searched the literature for studies measuring expression of *PIG* genes and *PGAP* genes in erythroid cells. Merryweather-Clarke et al.^26^ performed high resolution transcriptome analysis of cultured erythroid cells during maturation. Although not the main topic of that report, the supplemental data confirms no to very low expression of PGAP1 and absence of expression of PGAP5 in erythroid cells. As the function of PGAP5 is to remove EtNP on Man2 in the GPI cellular pathway, this suggests that EtNP on Man2 remains and is robustly expressed on RBCs and is the target of anti-Emm.

## Discussion

We show here, using family inheritance analysis and a genome wide search, that *PIGG* is responsible for the Emm blood group system. The absence of Emm on PNH cells had strongly suggested that Emm was on a GPI-anchored protein^4,8^, but the finding that Emm is a component of the anchor itself is an unexpected discovery. The presence of rare loss of function mutations in five unrelated and ethnically diverse families or individuals with Emm− phenotypes that correlate with the inheritance of unique mutations, paired with the role of this transferase in the biosynthesis of GPI-anchors, supports *PIGG* as the gene responsible. *PIGG* encodes ethanolamine phosphate transferase 2 which modifies the second mannose (Man2) of the GPI-anchor by adding ethanolamine phosphate (EtNP) (Fig. 5). Loss of function of *PIGG* in Emm− individuals would result in absence of EtNP on Man2 on the GPI-link (Fig. 5, H8). The finding that Emm is part of the GPI-anchor supports previous results showing that Emm− individuals have GPI-anchored proteins, yet anti-Emm does not bind to PNH RBCs which lack both GPI-anchors and their linked proteins.

The association of the Emm− phenotype and *PIGG* loss of function mutations may have important biological relevance. *PIGG* loss of function mutations have only recently been reported to be responsible for neurologic phenotypes including seizures, developmental delay, intellectual disability, and hypotonia^10,11^; this is an active area of investigation. The South Asian family members investigated here by WGS, and their physician, indicated there was no history of neurological or related symptoms. Regarding previous reports of individuals with Emm− RBC phenotypes, many are years if not decades old and were tested here from archived samples and medical histories are largely unknown. Only the first proband discovered, who was living in France and born in Madagascar, was reported to be suffering from an unknown neurological disease^3^. The Japanese proband (Proband 2) had a diagnosis of total blindness and renal carcinoma, and shown here the previously reported siblings with congenital neurologic symptoms have a Emm− phenotype. It is possible that the other Emm− probands had additional clinical phenotypes, but such information is generally not shared with the Transfusion Service whose main concern is ensuring transfusion compatibility. The association of this rare RBC phenotype with mutation in *PIGG*, suggests further medical history and evaluation of the probands and families reported here may shed important information on the biological effects of different rare *PIGG* mutations. It has been suggested that fibroblasts are more sensitive to pathogenic variants in GPI synthesis, as relates specifically to *PIGG*^11^, and are well suited to screen for GPI anchor deficiencies. Typing of the RBCs for Emm antigen, and or screening of the plasma for anti-Emm, would offer an accessible and rapid alternative for screening samples for pathogenic defects in GPI synthesis.

The prevalence of the Emm− phenotype in populations is of medical relevance for blood transfusion, as anti-Emm has been shown to be both naturally occurring and to cause hemolytic transfusion reactions. *PIGG* mutations are very rare, but there appears to be a large number of different *PIGG* loss of function variations in current genomic databases. The 2-bp deletion mutation, (rs771819481, c.2624_2625delTA) found in the South Asian family, is in gnomAD v3 with an allele frequency of 0.000014. However, the mutations found in the Japanese (deletion of Exons 2, 3), the European (deletion of Exons 7 thru 9), and the North African family (indel in Exon 3) have not been previously reported and not found in gnomAD SVs v.2.1. Homozygous loss of function mutations in *PIGG* are very rare, with only one found in gnomAD v3 [rs150259543, c.1515G > A, p.(Trp505Ter)]. This allele is found in 102/143,304 alleles, with a frequency of 0.000712. Rare heterozygous carriers are found in every ethnic group with exception of Amish and Ashkenazy Jewish, and with the highest allele number in Non-Finnish Europeans. The allele frequency of *PIGG* loss of function is 0.00107, which predicts a homozygous Emm− prevalence of 1/878,969 individuals.

Limitations to this study include the lack of in vitro expression studies and structural studies to determine the precise epitope that composes Emm on the GPI-anchor. Further studies are needed to define the structure of Emm. Nevertheless, the results of these five unrelated families already provide strong evidence linking *PIGG* with the phenotypic expression of the Emm antigen.

The findings here illustrate the power of using WGS with family cohorts to uncover the genetic basis of blood group systems. The observation that Emm− RBC phenotype reveals underlying mutations in *PIGG*, provides an approach to find other individuals with Emm null phenotypes, and may offer a potential diagnostic. The ISBT Red Cell Immunogenetics and Blood Group Terminology working party has designated Emm as the 42nd blood group system.

## Supplementary Information

Supplementary Information.

## Data Availability

The datasets generated during and/or analysed during the current study are available from the corresponding author on reasonable request. DNA sequences have been deposited to GenBank: Proband 1 with *PIGG* c.2624_2625delTA (MW355842), Proband 2 with *PIGG* Exons 2–3 deletion (MW355843), Proband 3 with *PIGG* Exons 7–9 deletion (MW355844), and Proband 4 with *PIGG* c.361-51_383delinsGACTT (MW355845), and Proband 5 with *PIGG* c.1640G > A (MZ596413).

## Code availability

The code generated during this study is available at Bitbucket https://bitbucket.org/lucare/emm_vcf_filter.
